# The Role of CXCR3 and Associated Chemokines in the Development of Atherosclerosis and During Myocardial Infarction

**DOI:** 10.3389/fimmu.2018.01932

**Published:** 2018-08-27

**Authors:** Veronika Szentes, Mária Gazdag, István Szokodi, Csaba A. Dézsi

**Affiliations:** ^1^Department of Cardiology, Petz Aladár County Teaching Hospital, Győr, Hungary; ^2^Gedeon Richter Plc., Budapest, Hungary; ^3^Heart Institute, Medical School, and Szentágothai Research Centre, University of Pécs, Pécs, Hungary

**Keywords:** inflammation, chemokine, I-TAC, atherosclerosis, coronary artery disease, myocardial infarction

## Abstract

The chemokine receptor CXCR3 and associated CXC chemokines have been extensively investigated in several inflammatory and autoimmune diseases as well as in tumor development. Recent studies have indicated the role of these chemokines also in cardiovascular diseases. We aimed to present current knowledge regarding the role of CXCR3-binding chemokines in the pathogenesis of atherosclerosis and during acute myocardial infarction.

## Introduction

Atherosclerosis is a chronic inflammatory disease, with immune cells and their effector molecules initiating and maintaining the progression of atherosclerotic lesion formation, accompanying and also precipitating acute coronary events and the following reparatory processes (1, 2). Chemotactic cytokines, or so-called chemokines have been shown to facilitate leukocyte migration during inflammatory responses to various stimuli, including their recruitment to the sites of atherosclerotic lesions (3).

Several chemokines have been associated with cardiovascular inflammatory changes. Chemokines CCL2, CCL5, CCL20, CXCL1, MIF (migration inhibitory factor), and CX_3_CL1 play a role in monocyte mobilization and recruitment (4). Monocyte binding to endothelial cells and their diapedesis into the subendothelial space is promoted by chemokine heterodimers CXCL4-CCL5. CXCL4 also affects monocyte differentiation into M4 macrophages, predominantly present in the adventitia and intima (5). Recruitment and survival of neutrophils is facilitated by CCL2, CCL3, CCL5, and CXCL1; (4) they also interact with CXCL4 (6) and CXCL12 (7).

**Figure 1:**
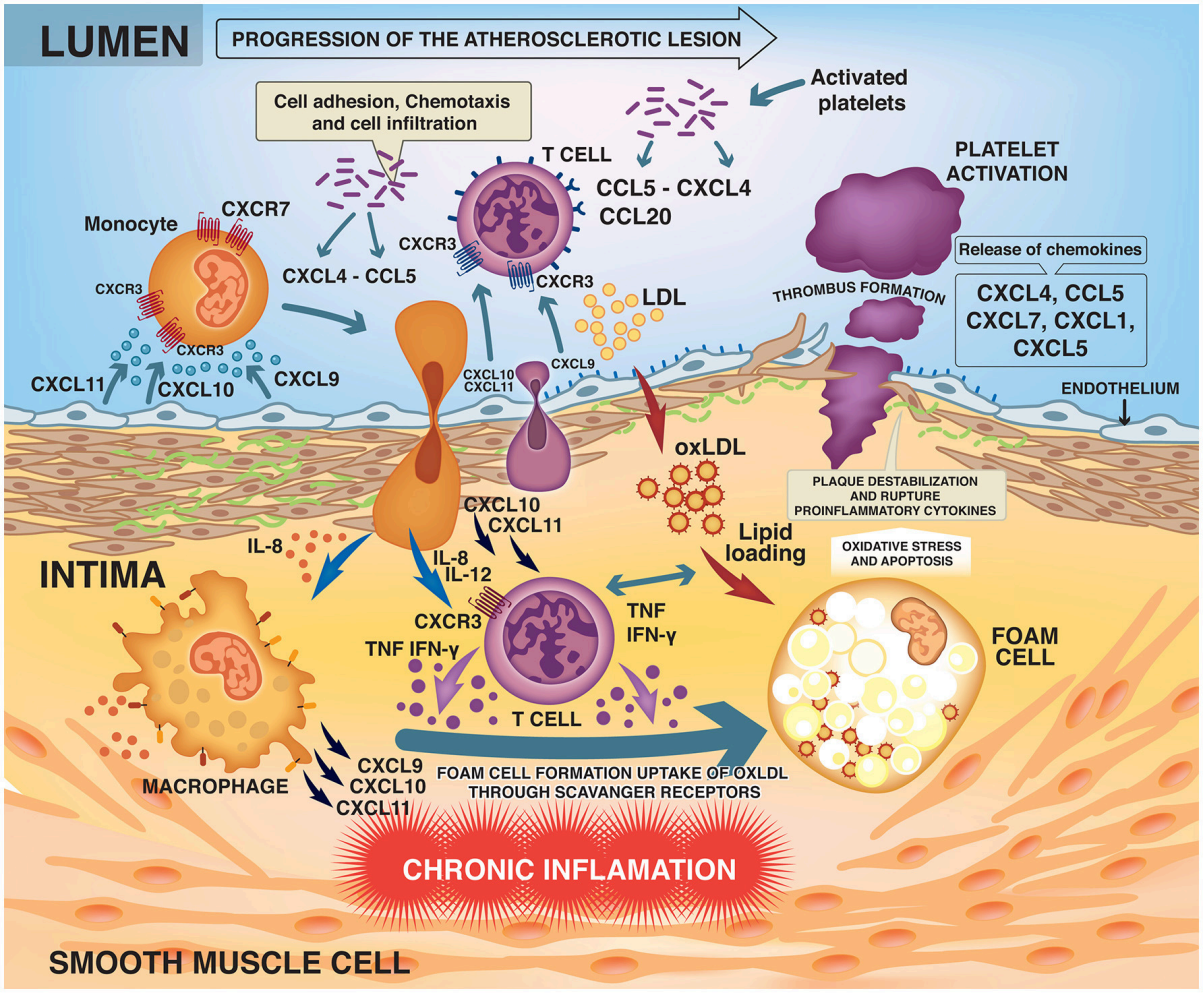
Development and progression of the atherosclerotic lesion.

Activated T lymphocytes (primarily Th1 cells) accumulate early and abundantly in the atherosclerotic lesions and are present in the plaques at all stages (3, 8). The Th1 cells recruited to the lesion recognize LDL as antigen and produce proinflammatory mediators such as interferon-gamma (IFN-γ) and tumor necrosis factor (TNF) (3, 8, 9). IFN-γ is the major proatherogenic cytokine, promoting local expression of adhesion molecules, cytokines and chemokines such as CXCL9, CXCL10, and CXCL11 and their main receptor CXCR3 by macrophages and endothelial cells (10). Chemokine signaling through CXCR3 facilitates recruitment and selective homing of active Th1 cells to the site of plaque development or rupture (Figure 1) (10–12).

The present review focuses on the role of the IFN-γ inducible chemokines and their receptor CXCR3 in the development of atherosclerosis and consequent coronary artery disease. Possible clinical implications of the presented findings are not entirely clear, but the currently available clinical studies suggest that this might be a promising area of intervention in the future of cardiovascular therapy and prevention (13).

## Biased signaling through CXCR3

CXCR3 is a 7-transmembrane spanning (7-TMS) G-protein-coupled cell surface receptor that allows functional selectivity on tissue, receptor as well as ligand levels (6). It binds three inflammatory chemokines CXCL9, CXCL10, and CXCL11 (14, 15). It was also shown to weakly bind CXCL4 (platelet factor 4), with questionable *in vivo* significance (16). CXCR3 has three alternative splice variants: CXCR3A, CXCR3B, and CXCR3Alt that activate different intracellular signaling pathways, depending also on the ligand they bind (14). For example, Gαi heterotrimeric G protein activation and β-arrestin 1 and 2 recruitment was shown after stimulation with CXCL10 and CXCL11 on CXCR3A, however on CXCR3B it was shown only after stimulation with CXCL11 in high doses and was not detectable on CXCR3Alt. ERK1/2 phosphorylation and receptor internalization occurred on all three variants after stimulation, its intensity and signal duration depending on the chemokine ligand and splice variant assessed (14).

Different chemokines binding to CXCR3 appear to have slightly different roles in T cell trafficking. CXCL10 is abundantly expressed by all atheroma-associated cells such as T cells and monocytes and is supposed to facilitate T cell retention within the lesion (15, 17). CXCL11 interacts with CXCR3 with higher affinity and is a stronger agonist, demonstrated by its ability to mobilize intracellular calcium and also chemotactic migration of CXCR3+ cells. It is not active on resting or naïve T cells suggesting that CXCL11 does not play a role under normal conditions only during IL-2 stimulated T cell response (17, 18). CXCL11 was shown to be the physiologic inducer of CXCR3 down-regulation on the cellular surface after T cell contact with IFN-activated endothelial cells (19). This might serve as an arrest signal for the activated T cells and lead to restraining inflammatory responses (8). Besides CXCR3, CXCL11 also binds to receptor CXCR7 (ACKR3), which may also be a possible regulation point for CXCR3-mediated responses (16, 20). CXCL11 also has an antagonistic effect on CCR5, counteracting its inflammatory activities in leukocyte activation (21).

Biased signaling on CXCR3 results in different effect of its ligands during inflammatory events. It seems that CXCL9 and CXCL10 promote inflammation through inducing T cell polarization into Th1/Th17 cells, while CXCL11 drives the development of regulatory T cells (Treg) cells which play a role in restraining inflammation (22). Based on the above, CXCR3 may be hypothesized to play a dual role by mediating both proinflammatory and anti-inflammatory pathways.

## CXCR3 binding chemokines in atheroma development

Experimental data demonstrated that targeted deletion or pharmacological inhibition of CXCR3 results in reduced plaque formation, which is accompanied by reduced recruitment of Th1 cells and increased migration of regulatory T-lymphocytes to lesions in apoE–/– mice (23, 24). In line, Apoe–/– /Cxcl10–/– mice showed reduced atherogenesis with enhanced numbers and activity of Treg cells (25). Moreover, antibody-mediated CXCL10 inhibition resulted in a more stable plaque phenotype in a vulnerable plaque mouse model (26).

High levels of IFN-γ induced chemokines CXCL9, CXCL10, and CXCL11 can be detected in human atheromas throughout all stages of plaque development (7). Niki et al. found elevated CLXCL10 levels to be associated with coronary atherosclerosis (27), while Segers et al. revealed a close correlation between high local concentrations of CXCL10 and unstable plaque characteristics by analyzing human carotid plaque specimens (26). CXCL4 and CXCL12 were also detected within atherosclerotic lesions (7, 28). CXCL12 was suggested to mediate anti-inflammatory action through neutrophil cells (7). CXCL4 is produced by platelets and plays a role in T cell-platelet interactions (29). Its levels were found to be correlated with the histological and clinical severity of atherosclerosis (28).

## CXCR3 binding chemokines in angina pectoris

There is an increased systemic inflammatory activity present in patients with coronary artery disease, characterized by an increased proportion of IFN-γ positive Th1 lymphocytes. In patients with stable angina pectoris, enhanced systemic expression of CXCL9, CXCL10, and CXCR3 can be observed. Interestingly, lower levels of these chemokines and CXCR3 were found in the peripheral cells of patients with acute coronary syndrome, which indicates a sequestering of circulating CXCR positive cells from blood to the site of infarction via an intense *in situ* release of these chemokines (10, 11). Plasma levels of CXCL12 are decreased in patients with stable and unstable angina compared with healthy controls. CXCL12 thus might have a protective effect in unstable angina through stabilizing the atherosclerotic plaque (7).

Other anti-inflammatory molecules known for their protective effect in cardiovascular diseases were found to influence T cell trafficking through the chemokine system. Adiponectin was shown to inhibit CXCR3 ligand production in macrophages, while heparin competes for binding with CXCL9, CXCL10, and CXCL11 on endothelial cells (30, 31).

## CXCR3 binding chemokines in myocardial infarction

It has been reported that CXCL10 and CXCR3 mRNA levels are up-regulated in the infarcted murine myocardium, with a marked increase in the number of CXCR3+/CD45+ leukocytes, CXCR3+/CD3+ T lymphocytes and CXCR3+ myofibroblasts (32, 33). Although CXCR3 is well-known to activate pro-inflammatory Th1 lymphocyte responses, deficiency of CXCR3 did not affect post-infarction cardiac remodeling (34). In contrast, Cxcl10–/– mice subjected to myocardial infarction were characterized by enhanced adverse ventricular remodeling, early expansion of the fibrotic scar, and increased neutrophil infiltration with marked reduction of recruitment of CXCR3 expressing leukocytes and T cells (33). Notably, CXCR3-independent proteoglycan signaling may mediate the anti-fibrotic effects of CXCL10 in the infarcted heart (34). In contrast to CXCL10, the role of CXCL9 and CXCL11 in infarct healing is not known.

Through receptor CXCR3, CXCL9, and CXCL10 promote T cell polarization into effector Th1/Th17 cells releasing pro-inflammatory mediators. Meanwhile, CXCL4 and CXCL11 promoted the differentiation of T cells into Treg1 cells, responsible for restraining the inflammatory response through IL-10, TGF-β and contact dependent pathways (22, 35, 36). Platelet surface expression of CXCR4 and CXCR7 receptors is elevated in acute coronary syndrome compared to stable angina. High CXCR7 levels are also associated with better improvement of left ventricular function after myocardial infarction. CXCR7 expression might contribute to regenerative function of platelets following acute coronary events (37).

Timely resolution of cardiac inflammatory responses plays a pivotal role in optimal tissue reparation (38, 39). Excessive, prolonged or inadequately contained inflammation can cause several complications such as cardiac rupture or dilatative ventricular remodeling and may lead to impaired cardiac function. Activation of pro-apoptotic pathways can cause unnecessary loss of cardiomyocytes and the extension of the inflammation to the non-infarcted area results in enhanced fibrosis and increased infarct size (38–40). Blockade of leukocyte related inflammatory mediators was shown to cause a marked reduction in infarct size and prevented the extension of ischemic cardiomyocyte injury following reperfusion in experimental studies (38).

During myocardial infarction, the dual role of CXCR3 in inflammatory processes might enable CXCR3+ cells to set off an appropriately rapid and robust inflammatory response in the beginning (1). Also, it might contribute to the timely resolution of symptoms by restraining the inflammation afterwards. It seems that the activation of the plaque rather than the degree of coronary stenosis precipitates ischemia and infarction. Endothelial erosion or plaque rupture was found to be responsible for the majority of coronary thrombotic events (1, 9) (Box 1).

Box 1 **Chemokines, CXCR3, and CXCL9 (Mig), CXCL10 (IP-10), and CXCL11 (I-TAC)**

**Chemokines**

Chemokines are a structurally related superfamily of more than 50 small signaling proteins (cytokines) that were originally named after their chemotactic effect on leukocytes. They all share a conserved cysteine motif in the mature sequence of the chemokines. Based on the number and arrangement of the N-terminal cysteine residues in this motif, chemokines can be divided into four families (CXC, CC, C, and CX3C) (41, 42). Besides regulating leukocyte migration and degranulation, chemokines take active part in a number of complex processes like angiogenesis or hematopoiesis and were found to participate in several diseases related to the immune system such as atherogenesis, multiple sclerosis, asthma, HIV-infection or cancer (7, 18, 41–43).

Chemokines bind to 7-TMS G-protein-coupled cell surface receptors. The activation of chemokine receptors can be followed by one of several signaling pathways, including inhibition of adenylate cyclase, activation of phosphoinositol 3-kinase, phospholipase C and D, protein kinase C and A, inositol triphosphate generation and transient calcium influx (44). More than 20 chemokine receptors have been discovered so far; their names mirror the nomenclature of chemokine family names (CXCR1-7, CCR1-10, etc.) (45).

**CXCR3**

CXCR3 is a chemokine receptor expressed by activated T lymphocytes, including CD4+ T helper 1 (Th1) cells, CD8+ cytotoxic T lymphocytes (CTL), and CD4+ and CD8+ memory T cells, as well as monocytes, M1 macrophages, natural killer (NK) cells, leukemic B-cells, eosinophils, mast cells, plasmocytoid dendritic cells, endothelial cells (ECs) and vascular smooth muscle cells (SMCs) (44, 46). Up-regulation of CXCR3 has been described in multiple sclerosis and transplant rejection (47). CXCR3 is also expressed by various tumor cells (48).

**CXCL9 (Mig), CXCL10 (IP-10), and CXCL11 (I-TAC)**

These three non-ELR chemokines are on the same branch of the phylogenetic tree and consequently share common characteristics. Their main receptor is CXCR3, but they can also act as antagonists for CCR3. They are constitutively expressed at low levels in normal tissues including thymus and spleen, where they are probably involved in activated (CXCR3+) T cell trafficking. Their expression is strongly induced by IFN-γ and they are produced in a wide variety of cell types, including atheroma-associated endothelial cells and macrophages (7, 17, 41, 44).

## Clinical experience with CXCR3 binding chemokines in ischemic heart disease

The clinical relevance of CXCR3 binding chemokines in ischemic heart disease is not fully understood. As summarized in Table 1, clinical studies to date aimed to find an association between plasma levels of different cytokines and several aspects of coronary events. It seems that complex patterns rather than individual changes in plasma chemokine levels might be associated with cardiovascular risk (50, 53, 59). Ardigo et al. found that when using a combined multimarker chemokine model (including CXCL10), serum concentrations of the chemokines were differentially regulated in individuals with clinical coronary artery disease compared with subjects with no such history. Their findings suggest that chemokine profile models using multiple chemokines may represent a strong signal of coronary artery disease with even higher specificity than traditional risk factors (49).

**Table 1:** Clinical studies with CXCR3 binding chemokines in coronary artery disease.

**Study**	**Molecules/** **Receptor**	***n***	**Disease/Intervention**	**Description**	**Results**
Ardigo et al. (49)	CXCL10 CCL11/eotaxin-1 CCL2/MCP-1 CCL3 CCL7 CCL8 CCL13 (CXCL8 and CCL5/RANTES not analyzed)	50 patients 48 controls	CAD, incident AMI	Cross-sectional study of a multidimensional approach, utilizing profiles of several inflammatory biomarkers.	Models using multiple chemokines more accurately distinguished cases and controls compared with models using traditional risk factors.
Rothenbacher et al. (50)	CXCL10 IL-8 RANTES/CCL5 MCP-1/CCL2 MIP-1α	312 patients 472 controls	Stable CAD	Case-control study investigating the association of chemokines with the risk of stable coronary heart disease.	Serum levels of CXCL10 and IL-8 were higher, and serum levels of RANTES were lower in CHD patients when compared with age- and gender-matched controls.
Fernandes et al. (11)	CXCL9 CXCL10 CXCR3 IL-12 IFN-γ	50 patients 10 controls	Stable or unstable angina pectoris	To explore whether this increase in Th1 activity could also be detected in circulating cells indicating a systemic activation.	Serum IL-12 and intracellular expression of IFN-γ were significantly elevated in patients with unstable angina. An enhanced expression of IFN-γ chemokines IP-10, Mig and CXCR3 in patients with stable angina was also observed.
Safa et al. (51)	CXCL10	300 patients 100 controls	Stable or unstable angina pectoris AMI	A comparative study to evaluate the CXCL10, CCL20 and CCL22 levels in patients with ischemic heart disease.	Serum levels of CXCL10 were significantly higher in patients with AMI, SA or UA as compared with the healthy control group.
PRIME (52)	CXCL10 RANTES/CCL5 MCP-1/CCL2 eotaxin-1/CCL11	621 patients 1242 controls	CAD	To quantify the association between systemic levels of chemokines with future coronary heart disease and to assess their usefulness for risk prediction.	None of the chemokines were independent predictors of CAD, either with respect to stable angina or to acute coronary syndrome.
MONICA/CORA Augsburg (53)	CXCL10 MCP-1/CCL2 IL-8	381 patients 1977 controls	CAD	To assess whether elevated systemic levels of these chemokines precede coronary events.	Elevated systemic levels of the chemokines MCP-1, IL-8, and CXCL10 precede CAHD but do not represent independent risk factors.
The Tromsø study (54)	CXCL10 apolipoprotein B/apolipoprotein A1 ratio kallikrein lipoprotein a matrix metalloproteinase 9 thrombospondin 4	419 patients 398 controls	AMI	To survey multiple protein biomarkers for association with the 10-year risk of incident AMI and identify a clinically significant risk model.	The protein biomarker model improved identification of 10-year AMI risk above and beyond traditional risk factors with 14% better allocation to either high or low risk group.
Ferdousie et al. (55)	CXCL10 CXCL12	80 patients	CAD/PTCA	To evaluate the potential correlation between serum levels of chemokines CXCL10 and CXCL12 and the degree of coronary artery occlusion.	A significant correlation between the serum levels of CXCL10 and CXCL12 and the severity of coronary artery occlusion was found.
Kawamura et al. (56)	CXCL10 MCP-1 CCR2 CCR5 CXCR2 CXCR3	55 patients 20 controls	CAD/PTCA	To investigate whether coronary stenosis is associated with a significant expression ofleukocyte CXCL10 –CXCR3.	Increased plasma concentrations of IP10 were accompanied by a compensatory decrease in the CXCR3 expression on lymphocytes, but not monocytes.
Ørn et al. (57)	CCL4 CXCL8 CXCL10 CXCL16 CCL3 CXCL7	42 patients	AMI/PCI	To assess the levels of selected chemokines during AMI and the subsequent 60 days.	After PCI, high levels of CCL4, CXCL16, CXCL10 and CXCL8 within the first week after PCI correlated positively with the degree of myocardial damage and infarct size after 2 months.
Koten et al. (58)	CXCL10	53 patients 20 controls	AMI/PCI stable angina pectoris	To examine the serum levels of CXCL10 in AMI.	The serum CXCL10 level was increased in AMI, and a higher level of serum CXCL10 before PCI may be informative regarding infarct size.
Keeley et al. (59)	CXCL1 CXCL5 CXCL8 CXCL9 CXCL10 CXCL11 CXCL12	156 patients	Coronary artery stenosis	To examine whether plasma levels of angiogenic and angiostatic chemokines are associated with of the presence and extent of coronary collaterals in patients with chronic ischemic heart disease.	Plasma chemokine concentrations are associated with the presence and extent of spontaneously visible coronary artery collaterals and may be mechanistically involved in their recruitment.
Kao et al. (60)	CXCL11 CCR5		Transplant CAD	To demonstrate that CXCL11 is involved in the pathogenesis of transplant CAD.	This study demonstrated a correlation between circulating CXCL11 chemokine levels and development of transplant CAD in humans.

In a large case-control study of 312 patients with coronary heart disease and 472 controls, a significant association of increased serum CXCL10 was found with the risk of coronary heart disease. Higher CXCL10 levels were also found to be independently correlated with established laboratory risk markers of coronary heart disease such as acute-phase proteins and inflammatory cytokines (50).

In patients with stable angina pectoris, Fernandes et al. found significantly higher levels of CXCL9, CXCL10, and CXCR3 compared to healthy controls (11). In patients with unstable angina, increased inflammatory activity was confirmed compared to stable angina patients by elevated high sensitivity C-reactive protein and serum amyloid A protein levels. However, the levels of CXCL9, CXCL10, and CXCR3 remained low in patients with unstable angina, comparable to the control group and significantly lower than in patients with stable angina. The authors suggested local release and intense uptake of these molecules by circulating leukocytes migrating to the site of active inflammation, which would explain their lower levels in the peripheral blood. Blood samples were drawn within 48 h of the index consultation of the unstable patients, and it was hypothesized that samples taken in a different time frame might capture serum elevations in CXCR3 and related chemokines (11).

Safa et al. (51) in a larger study in 260 patients and 100 healthy controls managed to capture elevated CXCL10 levels in patients with unstable angina. In this study the serum levels of CXCL10 were measured at the time of admission and were found to be elevated both in patients with stable and unstable angina pectoris. CXCL10 was also elevated in acute myocardial infarction, measured 3–5 days after admission. The study also confirmed the correlation of tradition risk factor with CXCL10, as mean serum levels of CXCL10 in patients with hypertension, dyslipidemia, obesity, diabetes and smoking were significantly higher as compared to the control group (51).

While elevated serum CXCL10 was found to be significantly associated with increased risk of coronary heart disease, it was not an independent risk factor for future coronary events in population-based case-control studies (52, 53). CXCL10 modestly correlated with traditional cardiovascular risk factors in the PRIME study (49). Age was found to be the strongest positive confounder in the MONICA/CORA Augsburg cohort, with the levels of circulating immune mediators increasing with age (52). The investigators of the Tromsø Study found that higher CXCL10 levels were protective for women when assessing the 10-year risk of incident myocardial infarction. In the multivariable model, the composite risk of 6 biomarkers including CXCL10 improved the traditional risk factor model by 14% (54).

A significant correlation was found between elevated serum CXCL10 and CXCL12 levels and the severity of coronary artery occlusion in patients with coronary heart disease who underwent PTCA (55). In patients with restenosis after PTCA, decreased concentrations of CXCL10 were followed by the decrease of CXCR3 expression on lymphocytes but not monocytes, suggesting a possible role of CXCL10 signaling on monocytes in neointimal hyperplasia in patients with restenosis (56).

CXCL8, CXCL10, and CXCL16 were found to be correlated with maximum troponin T levels, infarct size and impaired myocardial function assessed by cardiac magnetic resonance in patients after successful PCI (57). Serum CXCL10 level before PCI also proved to be an independent predictor of cumulative CK release and was negatively correlated with infarct size, as indicated by peak CK and CK-MB enzymes (58).

Better clinical outcome was found to be associated with recruitment of coronary collaterals (61). This form of vascular remodeling was shown to be accompanied by alterations in chemokine levels (59). Higher levels of angiogenic ligands CXCL5, CXCL8, and CXCL12 indicate the presence of collaterals, while the concentration of the angiostatic CXCL11 was associated with their absence. The higher extent of collateralization was associated with increased CXCL1 and decreased CXCL9, CXCL10, and CXCL11 (59).

Several chemokines have been linked to the development of acute transplant rejection episodes and transplant coronary artery disease in animals and also in human studies (60). Following heart transplantation, elevated CXCL11 levels have shown an association with the development of severe transplant coronary artery disease (60). CXCR3 ligands have also been studied in patients with left ventricular dysfunction and heart failure (62–64). Circulating levels of CXCL9, CXCL10, and CXCL11 were increased in subclinical as well as symptomatic left ventricular dysfunction, reaching statistical significance only in symptomatic patients (62). Addition of these CXCR3 ligands to established risk factors significantly improved the risk prediction models for left ventricular dysfunction (63). In a pilot study by Altara et al. levels of CXCL10 positively correlated with the severity of heart failure, especially in patients with advanced heart failure (64). Also, higher systemic levels of CXCL10 have been demonstrated to be independent risk factors for ischemic stroke (52).

## Conclusions

The chemokine network specifically directs the trafficking of immune cells in homeostasis and during inflammation. Excessive or inappropriate chemokine expression can lead to unnecessary leukocyte recruitment typical for autoimmune or allergic diseases. Chemokines have been extensively studied in diseases associated with T cell mediated inflammatory responses like multiple sclerosis, asthma bronchiale, AIDS and also in patients with transplant rejection (47, 60, 65).

Inflammatory processes in ischemic heart disease involve intense chemokine signaling from the forming of the atherosclerotic plaque and plaque destabilization to all phases of acute coronary events and infarct healing (36). IFN-γ inducible chemokines CXCL9, CXCL10, and CXCL11 attract activated T cells through CXCR3 receptor to the site of infarction. Modulation of their action might prevent the excessive recruitment of leukocytes to sites of inflammation and consequently influence the clinical outcome of the disease (47).

CXCR3 binding chemokines might be promising biomarkers for the risk assessment of coronary heart disease. Chemokine levels however have a short half-life and may have high intraindividual variability; (52) this results in difficulties in estimating the best sampling time and may generate conflicting clinical results.

CXCL10 is the most extensively studied of the three chemokines in the clinical setting of ischemic heart disease; less is known about the role of CXCL9 and CXCL11. New clinical studies are needed to fill in the gaps and properly map the role of alterations in chemokine levels in coronary artery disease and during acute coronary events.

## Author contributions

All authors listed have made a substantial, direct and intellectual contribution to the work, and approved it for publication.

### Conflict of interest statement

The authors declare that the research was conducted in the absence of any commercial or financial relationships that could be construed as a potential conflict of interest.
